# Serum FABP5 concentration is a potential biomarker for residual risk of atherosclerosis in relation to cholesterol efflux from macrophages

**DOI:** 10.1038/s41598-017-00177-w

**Published:** 2017-03-16

**Authors:** Masato Furuhashi, Masatsune Ogura, Megumi Matsumoto, Satoshi Yuda, Atsuko Muranaka, Mina Kawamukai, Akina Omori, Marenao Tanaka, Norihito Moniwa, Hirofumi Ohnishi, Shigeyuki Saitoh, Mariko Harada-Shiba, Kazuaki Shimamoto, Tetsuji Miura

**Affiliations:** 1 Department of Cardiovascular, Renal and Metabolic Medicine, Sapporo Medical University School of Medicine, Sapporo, Japan; 2 Department of Molecular Innovation in Lipidology, National Cerebral and Cardiovascular Center Research Institute, Suita, Japan; 3 Devision of Cardiology, Cardiovascular Center, Teine Keijinkai Hospital, Sapporo, Japan; 4 Department of Public Health, Sapporo Medical University School of Medicine, Sapporo, Japan; 5 Department of Nursing, Division of Medical and Behavioral Subjects, Sapporo Medical University School of Health Sciences, Sapporo, Japan; 6 Japan Health Care College, Sapporo, Japan

## Abstract

Cholesterol efflux capacity (CEC) from macrophages, the first step in the reverse cholesterol transport pathway, is inversely associated with residual risk for atherosclerotic cardiovascular disease. Fatty acid-binding protein 4 (FABP4) and FABP5 are expressed in both adipocytes and macrophages and play significant roles in the development of insulin resistance and atherosclerosis. Both FABP4 and FABP5 are secreted from cells, and their circulating levels are associated with insulin resistance and atherosclerosis. We investigated the association between CEC and levels of FABP4 and FABP5 in 250 subjects without any medications. CEC was positively correlated with HDL cholesterol level and negatively correlated with concentrations of high-sensitivity C-reactive protein (hsCRP) and FABP5, but not FABP4. Multiple regression analysis demonstrated that FABP5 concentration was an independent predictor of CEC after adjustment of age, gender and levels of HDL cholesterol and hsCRP. In 129 of the 250 subjects who underwent carotid ultrasonography, mean intima-media thickness was negatively correlated with CEC and was positively correlated with concentrations of FABP4 and FABP5. In conclusion, in contrast to FABP4, circulating FABP5 is associated with decreased CEC and carotid atherosclerosis, suggesting that FABP5 level is a regulatory factor of CEC and a potential biomarker for residual risk of atherosclerosis.

## Introduction

Several risk factors for atherosclerotic cardiovascular disease, including dyslipidemia, diabetes mellitus, hypertension and smoking, have been reported^1^. Regarding dyslipidemia, the level of high-density lipoprotein (HDL) cholesterol is a potential candidate for reduction of residual risk after treatment with a statin for patients with a high level of high low-density lipoprotein (LDL) cholesterol^2, 3^. However, it has been shown that an increase in HDL level induced by cholesteryl ester transfer protein inhibitors failed to reduce the risk of recurrent cardiovascular events^4–6^. Quantitative measurement of HDL cholesterol is inadequate to assess the impact of HDL cholesterol on cardiovascular events, and improving the quality of HDL would be a better therapeutic target than simply raising HDL cholesterol level. We and others previously reported that cholesterol efflux capacity (CEC) from macrophages as an HDL function, which represents the first step of the reverse cholesterol transport pathway, is inversely associated with residual risk for atherosclerotic cardiovascular disease even after adjustment of HDL cholesterol level^7–10^, providing supportive evidence for the significance of HDL functionality over simple measurement of HDL cholesterol level.

Fatty acid-binding proteins (FABPs) are a family of intracellular lipid chaperones and they are approximately 14–15-kDa predominantly cytosolic proteins that regulate lipid trafficking and responses in cells^11–13^. Among FABPs, fatty acid-binding protein 4 (FABP4) and fatty acid-binding protein 5 (FABP5) are expressed in both adipocytes and macrophages. Previous studies using FABP4- and FABP5-deficient mice demonstrated that both FABP4 and FABP5 play significant roles in the development of insulin resistance, diabetes mellitus and atherosclerosis^14–19^. We previously demonstrated that inhibition of FABP4 in cells would be a novel therapeutic strategy against insulin resistance, diabetes mellitus and atherosclerosis^20^.

FABP4 is secreted from adipocytes in association with lipolysis via a non-classical secretion pathway^21, 22^, though there are no typical secretory signal peptides in the sequence of FABP4^11^. It has recently been shown that FABP4 is also secreted from macrophages^23^. Elevated circulating FABP4 level is associated with obesity, insulin resistance, type 2 diabetes mellitus, dyslipidemia, hypertension, renal dysfunction, cardiac dysfunction, atherosclerosis and cardiovascular events^23–34^. Circulating FABP4 has recently been reported to act as an adipokine for the development of insulin resistance^21^, and neutralization of FABP4 with an antibody to FABP4 could be a feasible approach for the treatment of diabetes mellitus^35^. On the other hand, secretome analyses showed that FABP5 is secreted from cells^36–39^, though the mechanism remains unclear. It has been shown that circulating FABP5 level is associated with several components of metabolic syndrome, including atherosclerosis of carotid and coronary arteries^26, 40, 41^.

However, little is known about the link between CEC and levels of FABP4 and FABP5 regarding the development of atherosclerosis. In the present study, we investigated the cross-sectional association between CEC, circulating levels of FABP4 and FABP5 and intima-media thickness (IMT), a marker of carotid atherosclerosis assessed by using carotid ultrasonography, in a general population who had not regularly taken any medications.

## Results

### Basal characteristics of the studied subjects with no medication

Characteristics of the 250 recruited subjects with no medication (male/female: 88/162) are shown in Table 1. Mean age, BMI and waist circumference of the recruited subjects were 61 ± 14 years, 22.7 ± 3.5 kg/m^2^ and 82.6 ± 10.3 cm, respectively. Male subjects had significantly larger BMI and waist circumference and had higher levels of diastolic blood pressure, triglycerides, fasting glucose, insulin, HOMA-R, HbA1c, BUN, creatinine, uric acid, AST, ALT, γGTP and hsCRP and lower levels of pulse rate, total cholesterol, LDL cholesterol, HDL cholesterol and FABP4 than did female subjects. No significant difference in age, systolic blood pressure, eGFR, BNP, FABP5 or CEC was found between the male and female subjects.

**Table 1:** Characteristics of the studied subjects without medication (n = 250).

	Total	Male	Female	P
n	250	88	162	
Age (years)	61 ± 14	62 ± 15	61 ± 14	0.362
Body mass index (kg/m^2^)	22.7 ± 3.5	23.5 ± 3.7	22.2 ± 3.3	0.007
Waist circumference (cm)	82.6 ± 10.3	85.6 ± 10.3	81.0 ± 10.0	0.001
Systolic blood pressure (mmHg)	133 ± 23	135 ± 21	131 ± 24	0.178
Diastolic blood pressure (mmHg)	76 ± 12	78 ± 12	75 ± 11	0.041
Pulse rate (beats/min)	71 ± 10	69 ± 12	72 ± 9	0.042
Biochemical data				
Total cholesterol (mg/dl)	204 ± 34	192 ± 31	210 ± 33	<0.001
LDL cholesterol (mg/dl)	122 ± 29	115 ± 29	126 ± 28	0.004
HDL cholesterol (mg/dl)	68 ± 19	60 ± 19	73 ± 17	<0.001
Triglycerides (mg/dl)	85 (64–119)	98 (73–153)	78 (56–100)	<0.001
Fasting glucose (mg/dl)	91 (86–97)	94 (88–103)	90 (86–96)	0.009
Insulin (µU/ml)	4.6 (3.4–6.5)	4.9 (3.4–7.1)	4.5 (3.4–6.2)	0.046
HOMA-R	1.06 (0.75–1.57)	1.16 (0.76–1.71)	0.99 (0.74–1.45)	0.041
HbA1c (%)	5.4 ± 0.4	5.5 ± 0.4	5.4 ± 0.4	0.033
Blood urea nitrogen (mg/dl)	15 ± 4	16 ± 5	15 ± 4	0.002
Creatinine (mg/dl)	0.7 ± 0.2	0.8 ± 0.2	0.7 ± 0.1	<0.001
eGFR (ml/min/1.73 m^2^)	72.8 ± 14.0	74.1 ± 13.9	72.1 ± 14.0	0.284
Uric acid (mg/dl)	4.7 ± 1.2	5.7 ± 1.3	4.5 ± 0.9	<0.001
AST (IU/l)	22 (19–26)	24 (20–29)	21 (18–24)	<0.001
ALT (IU/l)	18 (14–24)	22 (17–33)	16 (13–20)	<0.001
γGTP (IU/l)	19 (15–32)	30 (20–46)	17 (14–23)	<0.001
BNP (pg/ml)	16.6 (11.0–30.1)	13.3 (6.7–23.5)	20.7 (13.4–31.0)	0.613
hsCRP (mg/dl)	0.03 (0.02–0.07)	0.04 (0.02–0.11)	0.03 (0.02–0.06)	0.002
FABP4 (ng/ml)	11.4 (7.4–16.0)	8.6 (5.7–12.9)	12.9 (8.9–16.9)	<0.001
FABP5 (ng/ml)	1.5 (1.0–2.0)	1.5 (1.1–2.1)	1.4 (1.0–2.0)	0.265
Cholesterol efflux capacity	0.8 ± 0.1	0.8 ± 0.1	0.8 ± 0.1	0.240

Variables are expressed as number, means ± SD or medians (interquartile ranges).

AST, Aspartate transaminase; ALT, Alanine transaminase; BNP, brain natriuretic peptide.

eGFR, estimated glomerular filtration rate; FABP4, fatty acid-binding protein 4; FABP5, fatty acid-binding protein 5.

γGTP, γ-glutamyl transpeptidase; hsCRP, high-sensitivity C-reactive protein.

### Correlations of cholesterol efflux capacity and FABP5 level with clinical parameters

**Figure 1:**
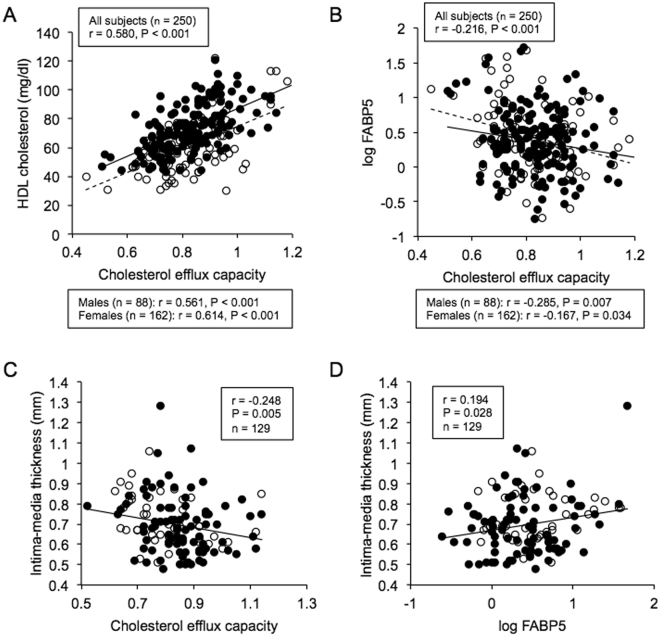
Correlations between cholesterol efflux capacity, levels of HDL cholesterol and FABP5 and carotid atherosclerosis. (**A**,**B**) HDL cholesterol level (**A**) and log FABP5 (**B**) were plotted against cholesterol efflux capacity in each subject (n = 250). Open circle and broken regression line: males (n = 88), closed circle and solid regression line: females (n = 162). (**C**,**D**) Mean intima-media thickness (IMT) was plotted against cholesterol efflux capacity (**C**) and log FABP5 (**D**) in each subject who underwent carotid ultrasonography (n = 129). Open circle: males (n = 44), closed circle: females (n = 85).

As shown in Table 2, CEC was positively correlated with levels of total cholesterol, HDL cholesterol (r = 0.580, P < 0.001) (Fig. 1A) and AST and was negatively correlated with age, systolic blood pressure and levels of triglycerides, fasting glucose, insulin, HOMA-R, HbA1c, BNP and hsCRP. A significantly negative correlation was found between CEC and concentration of FABP5 (r = −0.216, P < 0.001) (Fig. 1B) but not between CEC and concentration of FABP4 (r = −0.004, P = 0.945).

**Table 2:** Correlation analysis for cholesterol efflux capacity (n = 250).

	r	P
Age	−0.200	0.002
Body mass index	−0.115	0.070
Waist circumference	−0.122	0.055
Systolic blood pressure	−0.169	0.007
Diastolic blood pressure	−0.045	0.481
Pulse rate	0.071	0.274
Total cholesterol	0.261	<0.001
LDL cholesterol	−0.035	0.578
HDL cholesterol	0.580	<0.001
log Triglycerides	−0.231	<0.001
log Fasting glucose	−0.148	0.020
log Insulin	−0.190	0.003
log HOMA-R	−0.203	0.001
HbA1c	−0.134	0.035
Blood urea nitrogen	−0.039	0.537
Creatinine	−0.107	0.091
eGFR	0.109	0.085
Uric acid	0.001	0.991
log AST	0.146	0.021
log ALT	0.078	0.217
log γGTP	0.050	0.429
log BNP	−0.151	0.017
log hsCRP	−0.262	<0.001
log FABP4	−0.004	0.945
log FABP5	−0.216	<0.001

AST, Aspartate transaminase; ALT, Alanine transaminase.

BNP, brain natriuretic peptide; eGFR, estimated glomerular filtration rate.

FABP4, fatty acid-binding protein 4; FABP5, fatty acid-binding protein 5.

γGTP, γ-glutamyl transpeptidase; hsCRP, high-sensitivity C-reactive protein.

Serum FABP5 level was positively correlated with age, systolic blood pressure and levels of fasting glucose, HOMA-R, HbA1c, BUN, creatinine, uric acid, AST, ALT, BNP, hsCRP and FABP4 (r = 0.174, P = 0.006) and was negatively correlated with level of eGFR (r = −0.307, P < 0.001) and CEC (r = −0.216, P < 0.001) (Table 3).

**Table 3:** Correlation analysis for log FABP5 (n = 250).

	r	P
Age	0.327	<0.001
Body mass index	0.091	0.151
Waist circumference	0.111	0.079
Systolic blood pressure	0.139	0.029
Diastolic blood pressure	0.000	1.000
Pulse rate	0.004	0.951
Total cholesterol	−0.041	0.521
LDL cholesterol	0.002	0.981
HDL cholesterol	−0.111	0.079
log Triglycerides	0.111	0.081
log Fasting glucose	0.205	0.001
log Insulin	0.115	0.069
log HOMA-R	0.147	0.021
HbA1c	0.152	0.016
Blood urea nitrogen	0.226	<0.001
Creatinine	0.260	<0.001
eGFR	−0.307	<0.001
Uric acid	0.154	0.015
log AST	0.187	0.003
log ALT	0.134	0.035
log γGTP	0.095	0.136
log BNP	0.156	0.013
log hsCRP	0.157	0.015
log FABP4	0.174	0.006
Cholesterol efflux capacity	−0.216	<0.001

AST, Aspartate transaminase; ALT, Alanine transaminase.

BNP, brain natriuretic peptide; CEC, cholesterol efflux capacity.

eGFR, estimated glomerular filtration rate.

FABP4, fatty acid-binding protein 4; FABP5, fatty acid-binding protein 5.

γGTP, γ-glutamyl transpeptidase; hsCRP, high-sensitivity C-reactive protein.

Multiple regression analysis showed that CEC was independently associated with gender and levels of HDL cholesterol, hsCRP and FABP5, explaining a total of 37.8% of the variance in this measure (R^2^ = 0.378, AIC = 420.8) (Table 4). On the other hand, CEC was an independent predictor of FABP5 concentration after adjustment of age, gender and eGFR, explaining a total of 15.7% of the variance in this measure (R^2^ = 0.157, AIC = 293.9) (Table 4).

**Table 4:** Multiple regression analyses for cholesterol efflux capacity and log FABP5 (n = 250).

	Cholesterol efflux capacity		log FABP5	
	t	P	t	P
Age	−0.21	0.834	2.68	0.008
Gender (Male)	3.29	0.001	1.14	0.255
HDL cholesterol	10.20	<0.001	—	—
log hsCRP	−2.49	0.013	—	—
log FABP5	−2.20	0.029	—	—
eGFR	—	—	−2.76	0.006
Cholesterol efflux capacity	—	—	−2.54	0.012

R^2^ = 0.378, AIC = 420.8 (for Cholesterol efflux capacity); R^2^ = 0.157, AIC = 293.9 (for log FABP5).

FABP5, fatty acid-binding protein 5; hsCRP, high-sensitivity C-reactive protein.

### Associations of carotid atherosclerosis with cholesterol efflux capacity and FABP5 level

Among the recruited subjects, a total of 129 applicants (male/female: 44/85) underwent carotid ultrasonography, and basal characteristics are shown in Table S1. Clinical characteristics of the applicants were similar to those of the 250 enrolled subjects (Table 1, S1). No significant difference in mean IMT or mean stiffness parameter β was found between male and female subjects. Similar results were obtained for correlations of CEC or FABP5 level with clinical parameters (Table S2).

As shown in Table 5, mean IMT was negatively correlated with levels of HDL cholesterol and eGFR and with CEC (r = −0.248, P = 0.005) (Fig. 1C) and was positively correlated with age (r = 0.707, P < 0.001), systolic blood pressure and levels of HbA1c, BUN, creatinine, BNP, hsCRP, FABP4 (r = 0.178, P = 0.043) and FABP5 (r = 0.194, P = 0.028) (Fig. 1D). After adjustment of age and gender, mean IMT was independently associated with systolic blood pressure and CEC but not with levels of FABP4 and FABP5 (Table 5).

**Table 5:** Correlation and multiple regression analyses for mean IMT and log mean stiffness β (n = 129).

	Mean IMT				log Mean stiffness β			
	Correlation		Adjustment*		Correlation		Adjustment*	
	r	P	t	P	r	P	t	P
Age	0.707	<0.001	—		0.507	<0.001	—	
Body mass index	0.169	0.055	—		0.122	0.167	—	
Waist circumference	0.156	0.078	—		0.133	0.133	—	
Systolic blood pressure	0.418	<0.001	2.20	0.029	0.377	<0.001	2.46	0.015
Diastolic blood pressure	0.170	0.054	—		0.188	0.033	1.70	0.091
Pulse rate	−0.163	0.071	—		0.023	0.802	—	
Total cholesterol	−0.027	0.763	—		−0.037	0.678	—	
LDL cholesterol	0.128	0.149	—		0.027	0.762	—	
HDL cholesterol	−0.275	0.002	−1.13	0.261	−0.154	0.082	—	
log Triglycerides	0.171	0.052	—		0.119	0.181	—	
log Fasting glucose	0.170	0.054	—		0.181	0.040	1.15	0.253
log Insulin	0.089	0.318	—		0.040	0.653	—	
log HOMA-R	0.114	0.200	—		0.071	0.427	—	
HbA1c	0.193	0.029	0.71	0.480	0.167	0.058	—	
Blood urea nitrogen	0.243	0.006	0.43	0.665	0.140	0.113	—	
Creatinine	0.191	0.030	−0.17	0.866	0.095	0.285	—	
eGFR	−0.350	<0.001	0.24	0.814	−0.249	0.005	0.23	0.822
Uric acid	0.005	0.957	—		0.177	0.044	2.08	0.040
log AST	0.215	0.014	0.41	0.680	0.306	<0.001	2.49	0.014
log ALT	0.095	0.283	—		0.186	0.035	2.37	0.019
log γGTP	−0.033	0.712	—		0.048	0.592	—	
log BNP	0.366	<0.001	0.15	0.882	0.138	0.120	—	
log hsCRP	0.271	0.002	1.31	0.194	0.143	0.110	—	
log FABP4	0.178	0.043	−0.27	0.790	0.211	0.017	0.87	0.388
log FABP5	0.194	0.028	1.27	0.208	0.054	0.543	—	
CEC	−0.248	0.005	−2.09	0.039	−0.109	0.220	—	
Mean IMT	—	—	—		0.427	<0.001	1.27	0.208
log Mean stiffness β	0.427	<0.001	1.27	0.208	—	—	—	

AST, Aspartate transaminase; ALT, Alanine transaminase; BNP, brain natriuretic peptide; CEC, cholesterol efflux capacity.

eGFR, estimated glomerular filtration rate; FABP4, fatty acid-binding protein 4; FABP5, fatty acid-binding protein 5.

γGTP, γ-glutamyl transpeptidase; hsCRP, high-sensitivity C-reactive protein; IMT, intima-media thickness.

*Adjustment of age and gender in multiple regression analysis.

Mean stiffness parameter β, a marker of vascular stiffness, was negatively correlated with eGFR and positively correlated with mean IMT (r = 0.427, P < 0.001), age (r = 0.507, P < 0.001) and levels of systolic and diastolic blood pressures, fasting glucose, uric acid, AST, ALT and FABP4 (r = 0.211, P = 0.017) (Table 5). However, there was no significant correlation of mean stiffness β with CEC (r = −0.109, P = 0.220) or FABP5 level (r = 0.054, P = 0.543). After adjustment of age and gender, mean stiffness β was independently assocaited with systolic blood pressure and levels of uric acid, AST and ALT (Table 5).

## Discussion

The present study showed for the first time that serum FABP5 concentration was an independent negative predictor of CEC in connection with mean IMT in a general population who had not taken any medication, suggesting a link between circulating FABP5 and carotid atherosclerosis via reduction of cholesterol efflux in macrophages. Circulating FABP5 may directly regulate HDL function independently of HDL cholesterol level and decrease cholesterol efflux in macrophages. It has recently been shown that CEC was inversely associated with the incidence of cardiovascular events in a population-based cohort study^42^. Therapies targeting quality of HDL rather than quantity of HDL might be effective for prevention of atherosclerotic cardiovascular disease and prevention of recurrence, though it is necessary to determine whether CEC is associated with progression of atherosclerosis in subjects with no medication. Reduction of FABP5 level would be a novel therapeutic strategy for increasing CEC and preventing atherosclerotic cardiovascular disease.

**Figure 2:**
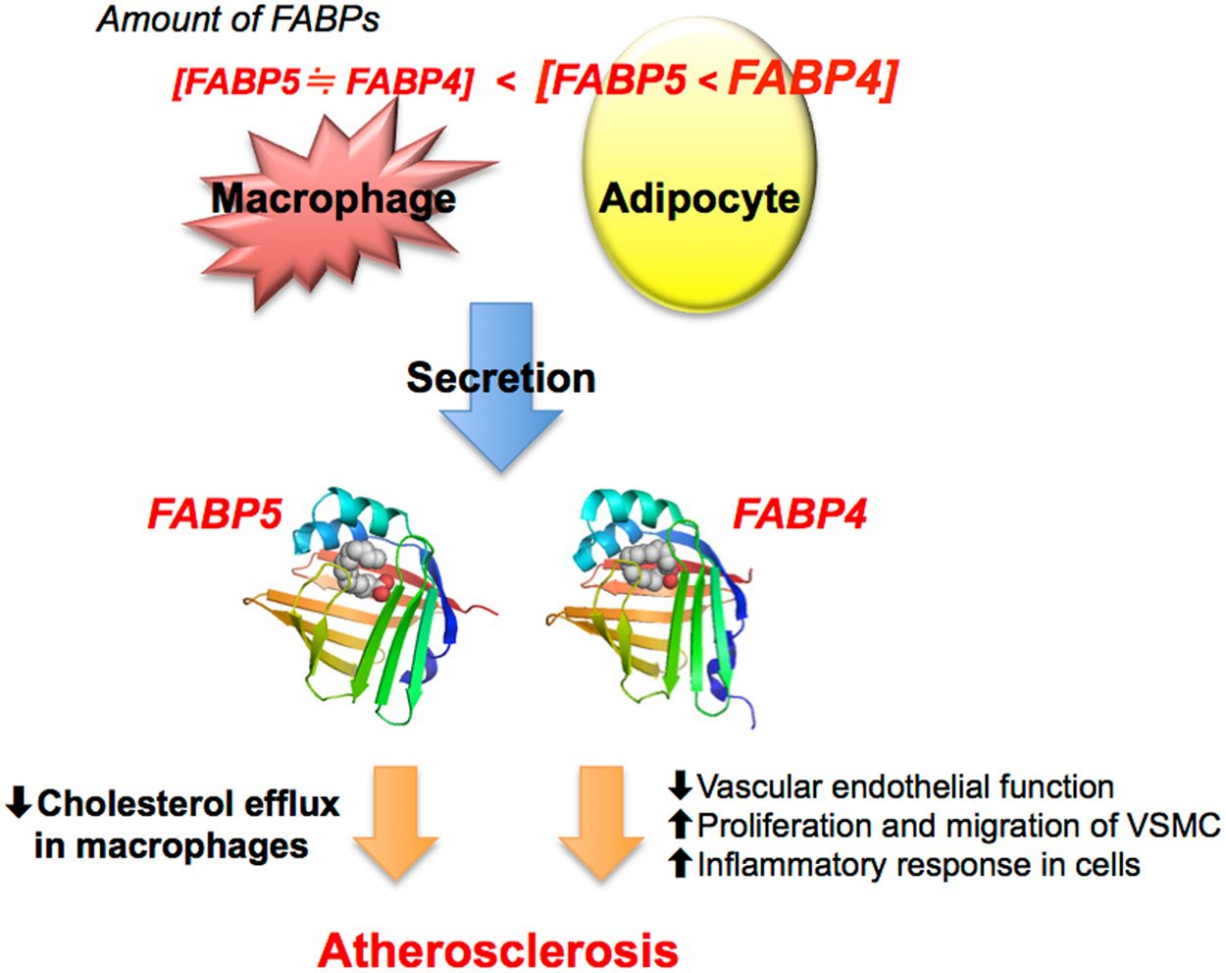
Putative mechanism of the development of atherosclerosis by secreted FABP4 and FABP5. The expression of FABP5 is about one-hundredth of that of FABP4 in adipose tissue, and the amount of FABP4 in adipocytes is about 10,000-fold larger than that in macrophages (ref. 11). The stoichiometry of FABP4 and that of FABP5 in macrophages are almost identical (ref. 15). Both FABP4 and FABP5 are secreted from adipocytes and macrophages (refs 21–23 and 39). Direct effects of exogenous FABP4 in various types of cells have been demonstrated. Treatment with recombinant FABP4 inhibited activation of endothelial nitric oxide synthase in vascular endothelial cells, increased proliferation/migration of vascular smooth muscle cells (VSMC) and induced inflammatory responses in macrophages, vascular endothelial cells and VSMC (ref. 23), leading to the development of atherosclerosis. In the present study, the level of FABP5, but not that of FABP4, was an independent negative predictor of cholesterol efflux capacity (CEC) as an HDL function, indicating that circulating FABP5 contributes to the development of atherosclerosis via reduction of CEC in macrophages. The mechanism of direct association between CEC and FABP5 level needs to be addressed in experimental models.

The two proteins FABP4 and FABP5 have 52% amino acid similarity and bind to various long-chain fatty acids with similar selectivity and affinity^11^. The expression of FABP5 is only about one-hundredth of that of FABP4 in adipose tissue^11^. Furthermore, circulating FABP5 level is detected at levels of about one tenth or less of FABP4 concentrations^26, 40, 41^, which was confirmed in the present study. However, it is notable that stoichiometry of FABP4 and that of FABP5 in macrophages are almost identical^15^. Previous studies using *in vitro* and *in vivo* experiments showed that FABP4 acts as an adipokine for the development of hepatic insulin resistance through increased hepatic glucose production^21^ and for the development of coronary atherosclerosis via induction of proinflammatory responses in macrophages, vascular smooth muscle cells and vascular endothelial cells^23^. Serum FABP4 level has also been reported to predict long-term cardiovascular events^32–34^. On the other hand, it has been reported that macrophage FABP5 deficiency suppresses atherosclerosis^17^. Interestingly, the impact of FABP5 on reduction of atherosclerosis was even greater than that of FABP4. In the present study, whereas mean IMT was negatively correlated with concentrations of FABP4 (r = 0.178, P = 0.043) and FABP5 (r = 0.194, P = 0.028), the level of FABP5, but not that of FABP4, was an independent predictor of CEC. A relatively high circulating FABP5 level derived from macrophages might be a reason for the difference in the impact of FABP4 and FABP5 levels on CEC. The putative mechanism of circulating FABP4 and FABP5 underlying the development of atherosclerosis is shown in Fig. 2.

FABP4 is secreted from adipocytes in association with lipolysis^21, 22^, and several drugs, including a statin^43^, omega-3 fatty acid ethyl esters^44^, a dipeptidyl peptidase-4 inhibitor^45^, a sodium glucose cotransporter 2 inhibitor^46^, a thiazolidinedione^47^ and angiotensin II receptor blockers^48, 49^, have been reported to modulate circulating FABP4 level. On the other hand, secretion of FABP5 remains to be elucidated. The mechanism of FABP5 secretion as well as direct association between CEC and FABP5 level needs to be addressed in experimental models. It is unclear whether FABP5 is loaded on HDL particles and directly regulates cholesterol efflux in macrophages. Identification of FABP5 using mass spectrometry analysis, such as in-depth proteomic analysis of purified HDL^50, 51^, would provide important information for predicting risks of atherosclerotic cardiovascular disease. Furthermore, the receptor for FABP5 remains unknown. It is unclear whether extracellular FABP5 is internalized into the cell or whether it acts by an intracellular signaling mechanism. A further understanding of the mechanism of FABP5 action may enable the development of new therapeutic strategies for atherosclerotic cardiovascular disease, such as neutralization of FABP5 and/or blockade of the FABP5 receptor, if any.

The present study has several limitations. First, the study was a cross-sectional design, which does not prove causal relations between serum level of FABP5, CEC and correlated biomarkers. A longitudinal study and interventional study are needed to clarify what underlies the relationship between FABP5 and CEC. Second, because the recruited subjects were only Japanese people, it is unclear whether the present findings can be generalized to other ethnicities. Third, the CEC assay quantifies not only one component of the reverse cholesterol transport pathway but also one property among several atheroprotective functions of HDL, including anti-inflammatory and anti-oxidative effects^3^. Lastly, CEC measurement is not standardized across laboratories. Therefore, values of CEC measured in the present study are not directly comparable to those measured in another laboratory using a different method.

In conclusion, in contrast to FABP4, circulating FABP5 is independently associated with both decreased CEC and carotid atherosclerosis, suggesting that FABP5 level is a regulatory factor of CEC and a potential biomarker for residual risk of atherosclerosis in relation to cholesterol efflux from macrophages. A further understanding of the mechanism underlying the link between FABP5 level and CEC may enable development of new therapeutic strategies for atherosclerotic cardiovascular diseases.

## Methods

### Study population

In the Tanno-Sobetsu Study, a study with a population-based cohort design in two rural towns, Tanno and Sobetsu, in Hokkaido, the northernmost island of Japan, a total of 617 Japanese subjects (male/female: 260/357, mean age: 66 ± 13 years) were recruited from residents of Sobetsu-town in 2011. Subjects who were being treated with any medications were excluded, and subjects who were not on any medication (n = 250, male/female: 88/162) were enrolled in the present study. This study conformed to the principles outlined in the Declaration of Helsinki and was performed with the approval of the Ethical Committee of Sapporo Medical University. Written informed consent was received from all of the study subjects.

Medical check-ups were performed between 06:00 h and 09:00 h after an overnight fast. After measuring anthropometric parameters, blood pressure was measured twice consecutively on the upper arm using an automated sphygmomanometer (HEM-907, Omron Co., Kyoto, Japan) with subjects in a seated resting position, and average blood pressure was used for analysis. Body mass index (BMI) was calculated as body weight (in kilograms) divided by the square of body height (in meters). Peripheral venous blood samples were obtained from study subjects after physical examination for complete blood count and biochemical analyses. Samples of the serum and plasma were analyzed immediately or stored at −80 °C until biochemical analyses.

### Measurements

Concentrations of FABP4 and FABP5 were measured using commercially available enzyme-linked immunosorbent assay kits for FABP4 (Biovendor R&D, Modrice, Czech Republic) and FABP5 (USCN Life Science, Houston, USA), respectively. The intra- and inter-assay coefficients of variation in the kits were <5%. According to the manufacturer’s protocol, no cross-reactivity of FABP4 or FABP5 with other FABP types was observed. Plasma glucose was determined by the glucose oxidase method. Fasting plasma insulin was measured by a chemiluminescent enzyme immunoassay method. Hemoglobin A1c (HbA1c) was determined by a latex coagulation method and was expressed in National Glycohemoglobin Standardization Program (NGSP) scale. Creatinine, blood urea nitrogen (BUN), uric acid, aspartate transaminase (AST), alanine aminotransferase (ALT), γ-glutamyl transpeptidase (γ-GTP) and lipid profiles, including total cholesterol, HDL cholesterol and triglycerides, were determined by enzymatic methods. LDL cholesterol level was calculated by the Friedewald equation. Brain natriuretic peptide (BNP) was measured using an assay kit (Shionogi & Co., Osaka, Japan). High-sensitivity C-reactive protein (hsCRP) was measured by a nephelometry method. Homeostasis model assessment of insulin resistance (HOMA-R), an index of insulin resistance, was calculated by the previously reported formula: HOMA-R = insulin (μU/ml) × glucose (mg/dl)/405. As an index of renal function, estimated glomerular filtration rate (eGFR) was calculated by an equation for Japanese^52^: eGFR (ml/min/1.73 m^2^) = 194 × creatinine^(−1.094)^ × age^(−0.287)^ × 0.739 (if female).

### Cholesterol efflux capacity

CEC was measured as previously reported^10^. In brief, apolipoprotein B (apoB)-depleted serum was obtained from whole serum by precipitating apoB-containing lipoproteins with a polyethylene glycol solution as described previously^53^. J774.1 cells, a murine macrophage cell line, were obtained from National Institute of Biomedical Innovation (Osaka, Japan) and were incubated with 0.33 μCi of ^3^H-cholesterol (Perkin-Elmer Analytical Sciences, MA, US) per milliliter for 24 h. Then the cells were washed with phosphate buffered saline, and an efflux medium containing 2.8% apolipoprotein B–depleted serum was added and left for 4 h. All steps were performed in the presence of 2 μg/ml Sandoz 58-035 (Santa Cruz Biotech), an acyl-coenzyme A:cholesterol acyltransferase inhibitor. Liquid scintillation counting (Perkin-Elmer Analytical Sciences, MA, US) was used to quantify the efflux of radioactive cholesterol from the cells. The quantity of radioactive cholesterol incorporated into cellular lipids was calculated by means of hexane and isopropanol extraction of control wells, which had not been exposed to the apoB-depleted serum. Percent efflux was calculated by the following formula: [(μCi of ^3^H-cholesterol in the medium containing 2.8% apoB-depleted serum - μCi of ^3^H-cholesterol in serum-free medium)/μCi of ^3^H-cholesterol in cells extracted before the efflux step] × 100. All assays were performed in duplicate. To correct for inter-assay variation across plates, a pooled serum control from eleven healthy volunteers was included on each plate, and values for serum samples from patients were normalized to the value of the pooled sample in subsequent analyses.

### Carotid ultrasonography

After medical check-ups and collection of urine and blood samples, carotid ultrasonographic examinations were performed in applicants by three well-experienced examiners certified by the Japan Society of Ultrasonics in Medicine, who were blinded to clinical data, using Vivid 9 (GE Health Care, Tokyo, Japan) equipped with a multifrequency 4- to 10-MHz linear-array transducer. Mean IMT of the far wall of the bilateral common carotid arteries was measured using commercially available semiautomated edge-detection software (IMT Option, General Electric Medical System, Milwaukee, WI, USA). The region of interest was placed from the beginning of carotid bulbs to a 2-cm proximal site in each common carotid artery. Stiffness parameter β of bilateral common carotid arteries was calculated from blood pressure and the dimension of common carotid arteries assessed by B-mode ultrasonography as stiffness parameter β = log (systolic blood pressure/diastolic blood pressure) × Dd/(Ds − Dd), where Dd and Ds are dimensions of the common carotid artery at end-diastole and end-systole, respectively. Mean values of IMT and stiffness parameter β in the bilateral common carotid arteries were averaged.

### Statistical analysis

Numeric variables are expressed as means ± SD for normal distributions or medians (interquartile ranges) for skewed variables. The distribution of each parameter was tested for its normality using the Shapiro-Wilk W test, and non-normally distributed parameters were logarithmically transformed for regression analyses. Comparison between two groups was done with the Mann-Whitney U test. The correlation between two variables was evaluated using Pearson’s correlation coefficient. Multivariate regression analysis was performed to identify independent determinants of CEC and FABP5 level using the variables with a significant and non-confounding correlation as independent predictors, showing the t-ratio calculated as the ratio of regression coefficient and standard error of regression coefficient and the percentage of variance in the object variables that the selected independent predictors explained (R^2^). Several models for independent determinants of CEC and FABP5 level were prepared by using all or different combinations of parameters as independent variables for calculation of both regression coefficients and Akaike’s Information Criterion (AIC). Among the candidate models, the best-fit model using AIC for each dependent variable was selected. Multiple regression analysis was also performed to identify the correlation of mean IMT or mean stiffness parameter β after adjustment of age and gender. A p value of less than 0.05 was considered statistically significant. All data were analyzed by using JMP 9 for Macintosh (SAS Institute, Cary, NC).

## Electronic supplementary material

Supplementary information
