# Supplementary material for: Serum FABP5 concentration is a potential biomarker for residual risk of atherosclerosis in relation to cholesterol efflux from macrophages
Source: Sci Rep. 2017 Mar 16;7:217. doi: 10.1038/s41598-017-00177-w (PMC5427929; doi:10.1038/s41598-017-00177-w)
Supplement: Supplementary file 1 — Supplementary information [file 41598_2017_177_MOESM1_ESM.pdf]

Supplementary information

Serum FABP5 concentration is a potential biomarker for residual risk of atherosclerosis in relation to cholesterol efflux from macrophages

Masato Furuhashi, Masatsune Ogura, Megumi Matsumoto, Satoshi Yuda, Atsuko Muranaka, Mina Kawamukai, Akina Omori, Marenao Tanaka, Norihito Moniwa, Hirofumi Ohnishi, Shigeyuki Saitoh, Mariko Harada-Shiba, Kazuaki Shimamoto, Tetsuji Miura

Table S1

Table S1. Characteristics of the studied subjects without medication who underwent carotid ultrasound (n = 129)

	Total	Male	Female	P
n	129	44	85	
Age (years)	62 ± 13	64 ± 13	61 ± 12	0.241
Body mass index (kg/m^2)	22.6 ± 3.5	24.0 ± 3.9	21.9 ± 3.1	0.003
Waist circumference (cm)	82.7 ± 10.4	87.1 ± 10.1	80.4 ± 9.9	0.001
Systolic blood pressure (mmHg)	131 ± 22	134 ± 21	130 ± 22	0.325
Diastolic blood pressure (mmHg)	76 ± 11	77 ± 11	75 ± 12	0.285
Pulse rate (beats/min)	70 ± 10	68 ± 13	72 ± 8	0.060
Biochemical data				
Total cholesterol (mg/dl)	207 ± 30	192 ± 28	214 ± 28	< 0.001
LDL cholesterol (mg/dl)	124 ± 27	116 ± 28	129 ± 26	0.014
HDL cholesterol (mg/dl)	70 ± 19	60 ± 20	75 ± 16	< 0.001
Triglycerides (mg/dl)	81 (65-109)	92 (74-153)	76 (62-100)	0.002
Fasting glucose (mg/dl)	91 (86-97)	95 (90-104)	90 (86-95)	< 0.001
Insulin (μU/ml)	4.4 (3.2-6.3)	5.2 (3.2-7.3)	3.9 (3.1-5.8)	0.065
HOMA-R	1.00 (0.71-1.48)	1.28 (0.72-1.85)	0.87 (0.69-1.27)	0.034
HbA1c (%)	5.4 ± 0.4	5.6 ± 0.4	5.4 ± 0.3	0.006
Blood urea nitrogen (mg/dl)	15 ± 4	17 ± 5	15 ± 4	0.010
Creatinine (mg/dl)	0.7 ± 0.2	0.9 ± 0.2	0.7 ± 0.1	< 0.001
eGFR (ml/min/1.73m^2)	72.8 ± 13.9	73.4 ± 14.8	72.5 ± 13.5	0.737
Uric acid (mg/dl)	4.8 ± 1.2	5.4 ± 1.3	4.4 ± 1.0	< 0.001
AST (IU/l)	24 (20-26)	25 (20-29)	21 (20-24)	0.039
ALT (IU/l)	18 (15-24)	22 (17-34)	17 (14-21)	< 0.001
γGTP (IU/l)	19 (15-30)	28 (19-46)	17 (14-24)	0.007
BNP (pg/ml)	16.4 (11.1-28.3)	13.8 (8.0-18.3)	20.3 (12.7-30.8)	0.699
hsCRP (mg/dl)	0.03 (0.02-0.07)	0.04 (0.02-0.15)	0.02 (0.01-0.05)	0.002
FABP4 (ng/ml)	11.6 (7.5-16.1)	9.1 (5.5-12.7)	13.5 (8.3-18.2)	0.009
FABP5 (ng/ml)	1.5 (1.2-2.1)	1.6 (1.2-2.1)	1.5 (1.0-2.0)	0.119
Cholesterol efflux capacity	0.8 ± 0.1	0.8 ± 0.1	0.9 ± 0.1	0.035
Carotid ultrasonographic data				
mean IMT (mm)	0.70 ± 0.14	0.72 ± 0.12	0.69 ± 0.14	0.182
mean Stiffness β	6.1 (4.5-8.3)	6.4 (4.6-8.4)	5.7 (4.5-8.3)	0.989

Variables are expressed as number, means ± SD or medians (interquartile ranges).
AST, Aspartate transaminase; ALT, Alanine transaminase; BNP, brain natriuretic peptide;
eGFR, estimated glomerular filtration rate; FABP4, fatty acid-binding protein 4; FABP5, fatty acid-binding protein 5;
γGTP, γ-glutamyl transpeptidase; hsCRP, high-sensitivity C-reactive protein; IMT, intima-media thickness.

Table S2

Table S2. Correlation analyses for CEC and log FABP5 (n = 129)

	CEC		log FABP5	
	r	P	r	P
Age	-0.164	0.063	0.157	0.076
Body mass index	-0.075	0.400	0.101	0.254
Waist circumference	-0.076	0.390	0.113	0.201
Systolic blood pressure	-0.142	0.108	-0.049	0.585
Diastolic blood pressure	-0.060	0.501	-0.125	0.158
Pulse rate	0.172	0.056	-0.055	0.547
Total cholesterol	0.263	0.003	0.129	0.144
LDL cholesterol	-0.083	0.350	-0.102	0.250
HDL cholesterol	0.555	< 0.001	-0.078	0.388
log Triglycerides	-0.311	< 0.001	0.012	0.893
log Fasting glucose	-0.100	0.259	0.173	0.050
log Insulin	-0.230	0.009	0.092	0.299
log HOMA-R	-0.232	0.008	0.117	0.186
HbA1c	-0.018	0.843	0.017	0.849
Blood urea nitrogen	-0.003	0.777	0.267	0.002
Creatinine	-0.160	0.071	0.330	0.001
eGFR	0.120	0.174	-0.293	0.001
Uric acid	-0.023	0.799	0.195	0.027
log AST	0.086	0.332	0.168	0.057
log ALT	0.028	0.753	0.069	0.439
log γGTP	0.008	0.929	0.063	0.475
log BNP	-0.086	0.332	0.062	0.485
log hsCRP	-0.214	0.016	0.037	0.682
log FABP4	0.082	0.356	0.088	0.321
log FABP5	-0.180	0.041	-	-

AST, Aspartate transaminase; ALT, Alanine transaminase;
BNP, brain natriuretic peptide; CEC, cholesterol efflux capacity;
eGFR, estimated glomerular filtration rate;
FABP4, fatty acid-binding protein 4; FABP5, fatty acid-binding protein 5;
γGTP, γ-glutamyl transpeptidase; hsCRP, high-sensitivity C-reactive protein.
